# Empagliflozin is associated with improvements in liver enzymes potentially consistent with reductions in liver fat: results from randomised trials including the EMPA-REG OUTCOME® trial

**DOI:** 10.1007/s00125-018-4702-3

**Published:** 2018-07-31

**Authors:** Naveed Sattar, David Fitchett, Stefan Hantel, Jyothis T. George, Bernard Zinman

**Affiliations:** 1 British Heart Foundation Glasgow Cardiovascular Research Centre, University of Glasgow, 126 University Place, Glasgow, G12 8TA UK; 2 St Michael’s Hospital, Division of Cardiology, University of Toronto, Toronto, ON Canada; 3 Boehringer Ingelheim International GmbH, Ingelheim, Germany; 4 Lunenfeld-Tanenbaum Research Institute, Mount Sinai Hospital, University of Toronto, Toronto, ON Canada

**Keywords:** Alanine aminotransferase, Aspartate aminotransferase, Non-alcoholic fatty liver disease, Sodium glucose cotransporter 2 inhibition, Type 2 diabetes mellitus

## Abstract

**Aims/hypothesis:**

In addition to beneficial effects on glycaemia and cardiovascular death, empagliflozin improves adiposity indices. We investigated the effect of empagliflozin on aminotransferases (correlates of liver fat) in individuals with type 2 diabetes.

**Methods:**

Changes from baseline alanine aminotransferase (ALT) and aspartate aminotransferase (AST) were assessed in the EMPA-REG OUTCOME^**®**^ trial (*n* = 7020), pooled data from four 24-week placebo-controlled trials (*n* = 2477) and a trial of empagliflozin vs glimepiride over 104 weeks (*n* = 1545). Analyses were performed using data from all participants and by tertiles of baseline aminotransferases.

**Results:**

In the EMPA-REG OUTCOME^**®**^ trial, mean ± SE changes from baseline ALT at week 28 were −2.96 ± 0.18 and −0.73 ± 0.25 U/l with empagliflozin and placebo, respectively (adjusted mean difference: −2.22 [95% CI −2.83, −1.62]; *p* < 0.0001). Reductions in ALT were greatest in the highest ALT tertile (placebo-adjusted mean difference at week 28: −4.36 U/l [95% CI −5.51, −3.21]; *p* < 0.0001). The adjusted mean difference in change in ALT was −3.15 U/l (95% CI −4.11, −2.18) with empagliflozin vs placebo at week 24 in pooled 24-week data, and −4.88 U/l (95% CI −6.68, −3.09) with empagliflozin vs glimepiride at week 28. ALT reductions were largely independent of changes in weight or HbA_1c_. AST changes showed similar patterns to ALT, but the reductions were considerably lower.

**Conclusions/interpretation:**

These highly consistent results suggest that empagliflozin reduces aminotransferases in individuals with type 2 diabetes, in a pattern (reductions in ALT>AST) that is potentially consistent with a reduction in liver fat, especially when ALT levels are high.

**Electronic supplementary material:**

The online version of this article (10.1007/s00125-018-4702-3) contains peer-reviewed but unedited supplementary material, which is available to authorised users.

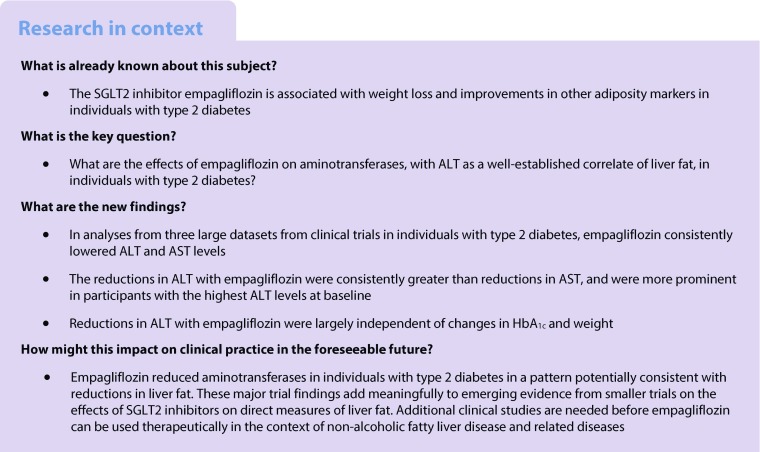



## Introduction

Non-alcoholic fatty liver disease (NAFLD) is estimated to affect more than half of those with type 2 diabetes [1–3]. The first recognisable stage of NAFLD is hepatic steatosis, when the fat content exceeds 5% of the liver volume [4]. Mechanistic insights suggest that ectopic liver fat is probably part of the pathogenic process in diabetes, contributing to hepatic insulin resistance, excess gluconeogenesis and higher fasting glucose levels [5]. Furthermore, hepatic steatosis due to NAFLD leads to, and is often clinically suspected by, increased levels of aminotransferases, with levels of alanine aminotransferase (ALT) exceeding those of aspartate aminotransferase (AST) [4]. Elevated ALT levels (typically >40–50 U/l) are common in individuals with type 2 diabetes [6–8] and for a given serum ALT, those with type 2 diabetes have more liver fat compared with BMI-, age- and sex-matched individuals without diabetes [9]. In a minority of individuals, hepatic steatosis progresses to non-alcoholic steatohepatitis (NASH), at which point liver inflammation and fibrosis necessitate monitoring and interventions to prevent progression to cirrhosis and other life-threatening sequelae [4]. In individuals with type 2 diabetes and elevated liver enzymes or NAFLD, improved glucose control may be important to delay the progression of liver disease [10, 11]. The glucose-lowering agents pioglitazone [12] and liraglutide [13] have been shown to reduce liver fat in individuals with NASH with and without type 2 diabetes, but the effects of newer agents on liver fat are less well studied.

Sodium–glucose cotransporter 2 (SGLT2) inhibitors reduce hyperglycaemia in individuals with type 2 diabetes by decreasing renal glucose reabsorption, thereby increasing urinary glucose excretion and lowering HbA_1c_ [14]. Empagliflozin, a highly selective SGLT2 inhibitor, was the first glucose-lowering agent to demonstrate a reduction in cardiovascular outcomes in individuals with type 2 diabetes and established cardiovascular disease, driven by a 38% reduction in cardiovascular death vs placebo [15]. Empagliflozin is associated with weight loss [15–22], probably due to osmotic diuresis and loss of calories in the urine [14, 23]. Nearly 90% of the weight loss with empagliflozin is due to a reduction in fat mass, with reductions in both abdominal visceral and subcutaneous adipose tissue [22]. This, in addition to improvements in other adiposity markers with empagliflozin [24], led us to hypothesise that empagliflozin may improve liver fat.

In the current analyses, we investigated the effects of empagliflozin on ALT and AST using several datasets collected in individuals with type 2 diabetes: the EMPA-REG OUTCOME^**®**^ trial in participants with type 2 diabetes and established cardiovascular disease [15], pooled data from four Phase III trials [16–19] and a head-to-head trial of empagliflozin vs glimepiride [22]. Given that empagliflozin therapy is associated with weight loss, we hypothesised that empagliflozin would lower ALT and AST, with greater reductions in ALT than AST, and that absolute reductions would be most evident in participants with higher ALT levels at baseline. We also investigated the contribution of weight loss and reduction in HbA_1c_ to improvements in ALT.

## Methods

### Study participants

The effect of empagliflozin on liver enzymes was analysed from three data sources. First, in the EMPA-REG OUTCOME^**®**^ trial (NCT01131676), participants with type 2 diabetes (HbA_1c_ 53–75 mmol/mol [7–9%] for drug-naive participants and 53–86 mmol/mol [7–10%] for those on stable glucose-lowering therapy), established cardiovascular disease, BMI ≤45 kg/m^2^, and eGFR (according to Modification of Diet in Renal Disease [MDRD]) ≥30 ml/min/1.73 m^2^ were randomised 1:1:1 to receive empagliflozin 10 mg, empagliflozin 25 mg or placebo once daily. Background glucose-lowering therapy was to remain unchanged for 12 weeks. After week 12, investigators were encouraged to adjust glucose-lowering therapy to achieve glycaemic control according to local guidelines. They were also encouraged to treat cardiovascular risk factors to achieve the best standard of care according to local guidelines throughout the trial. The trial was planned to continue until at least 691 participants had experienced an adjudicated event included in the primary outcome (three-point major adverse cardiovascular events: composite of cardiovascular death, non-fatal myocardial infarction and non-fatal stroke) [15].

Second, liver enzyme data from four Phase III trials in participants with type 2 diabetes were pooled. In these trials, participants with BMI ≤45 kg/m^2^, eGFR (MDRD) ≥30 ml/min/1.73 m^2^ (or ≥50 ml/min/1.73 m^2^ in the monotherapy trial) and HbA_1c_ ≥53 to ≤86 mmol/mol (≥7 to ≤10%) were randomised 1:1:1 to receive empagliflozin 10 mg, empagliflozin 25 mg or placebo once daily for 24 weeks as monotherapy (NCT01177813) [16], add-on to metformin (NCT01159600) [17], add-on to metformin plus sulfonylurea (NCT01159600) [18] or add-on to pioglitazone with or without metformin (NCT01210001) [19]. Rescue medication could be initiated if, after an overnight fast, a participant had a confirmed plasma glucose concentration of >13.3 mmol/l during weeks 1–12 or >11.1 mmol/l during weeks 12–24.

Finally, liver enzymes were analysed in the initial 104-week period of the EMPA-REG H2H-SU trial, in which participants with type 2 diabetes, BMI ≤45 kg/m^2^, HbA_1c_ 53–86 mmol/mol (7–10%) and eGFR (MDRD) ≥60 ml/min/1.73 m^2^ were randomised 1:1 to receive once-daily empagliflozin 25 mg or glimepiride 1–4 mg as add-on to metformin (NCT01167881) [22]. Glimepiride was initiated at a dose of 1 mg/day, with a recommendation for uptitration if fasting plasma glucose (assessed with home monitoring) was >6.1 mmol/l, to 2 mg/day at week 4, 3 mg/day at week 8 and 4 mg/day at week 12. Rescue medication could be initiated if, after an overnight fast, a participant had a confirmed blood glucose concentration of >13.3 mmol/l during weeks 1–12, >11.1 mmol/l during weeks 12–28 or >10.0 mmol/l (or HbA_1c_ >64 mmol/mol [>8%]) after week 28.

For all the trials that contributed data to these analyses, the clinical trial protocol was approved by the relevant institutional review boards and local independent ethics committees, and all of the trials were conducted in accordance with the Declaration of Helsinki and the International Conference on Harmonization guidelines for Good Clinical Practice. Participants provided signed and dated informed consent before entering each trial.

### ALT and AST measurements

In the EMPA-REG OUTCOME^**®**^ trial, samples for analysis of liver aminotransferases were taken during study visits at baseline and weeks 4, 12, 28 and 52, and then every 14 weeks until the end of the trial. In the 24-week placebo-controlled trials, samples for analysis of liver aminotransferases were taken during study visits at baseline and at weeks 12 and 24. In the initial 104 weeks of the EMPA-REG H2H-SU trial, samples for analysis of liver aminotransferases were taken during study visits at baseline and at weeks 12, 28, 52, 78 and 104. In all studies, analyses of liver aminotransferases were performed by a central laboratory.

### Analysis

Changes from baseline in ALT and AST were assessed using mixed-model repeated measures (MMRM) analyses, including baseline ALT or AST and baseline HbA_1c_ as linear covariates and region, baseline BMI, baseline eGFR, study, treatment, visit, baseline HbA_1c_ by visit interaction, baseline ALT or AST by visit interaction and visit by treatment interaction as fixed effects. The analysis of EMPA-REG OUTCOME^**®**^ data included an additional factor for the last week a participant could have had an ALT or AST measurement. MMRM analyses were based on observed cases, including values after initiation of rescue medication. In analyses of EMPA-REG OUTCOME^**®**^ and pooled data from the 24-week trials, analyses were performed for the pooled empagliflozin group and placebo. In each dataset, changes in ALT were analysed in all participants and in tertiles by ALT at baseline, and changes in AST were analysed in all participants and in tertiles by AST at baseline. Data are expressed as adjusted means (95% CI) or adjusted means ± SE.

In the EMPA-REG OUTCOME^**®**^ trial, the contribution of changes in weight or HbA_1c_ (individual and combined effects) to changes in ALT at weeks 28, 52 and 164 (median observation time) were assessed using MMRM analyses as described above, with additional factors for change in HbA_1c_ or weight and, for weight analyses only, baseline weight by visit interaction. These contribution analyses were performed for all participants.

## Results

### Participants

A total of 7020 participants with type 2 diabetes and established cardiovascular disease were treated in the EMPA-REG OUTCOME^**®**^ trial (2333 in the placebo group and 4687 in the pooled empagliflozin group). Baseline participant characteristics have been previously described [15]. In brief, 71% of participants were male, 72% were white and 22% were of Asian race, mean ± SD BMI was 30.6 ± 5.3 kg/m^2^, weight was 86.3 ± 18.9 kg and HbA_1c_ was 65 ± 98.3 mmol/mol (8.1 ± 0.85%). Mean ± SD ALT was 26.2 ± 15.3 U/l in the placebo group and 25.5 ± 13.8 U/l in the empagliflozin group, and AST was 22.9 ± 10.3 and 22.5 ± 9.6 U/l, respectively.

Data pooled from the 24-week trials included data from 2477 participants (825 in the placebo group and 1652 in the pooled empagliflozin group). Baseline participant characteristics in the individual trials have been previously described [16–19]. In the pooled dataset, 62% were male, 56% were of Asian race and 41% were white, baseline mean ± SD BMI was 29.0 ± 5.5 kg/m^2^, weight was 78.6 ± 18.8 kg and HbA_1c_ was 64 ± 9.3 mmol/mol (7.99 ± 0.85%). Mean ± SD ALT was 28.4 ± 17.2 U/l in the placebo group and 28.2 ± 15.7 U/l in the empagliflozin group, and AST was 23.1 ± 10.4 and 23.0 ± 9.7 U/l, respectively.

In the EMPA-REG H2H-SU trial, 1545 participants were treated (780 in the glimepiride group and 765 in the empagliflozin group). Baseline participant characteristics have been previously described [22]. In brief, 55% were male, 66% were white and 33% were of Asian race, mean ± SD BMI was 30.1 ± 5.3 kg/m^2^, weight was 82.8 ± 19.2 kg and HbA_1c_ was 63.0 ± 9.2 mmol/mol (7.92 ± 0.84%). Mean ± SD ALT was 31.2 ± 22.6 U/l in the glimepiride group and 31.9 ± 19.6 U/l in the empagliflozin group, and AST was 25.0 ± 12.8 and 24.7 ± 11.6 U/l, respectively.

### Changes in ALT

**Fig. 1:**
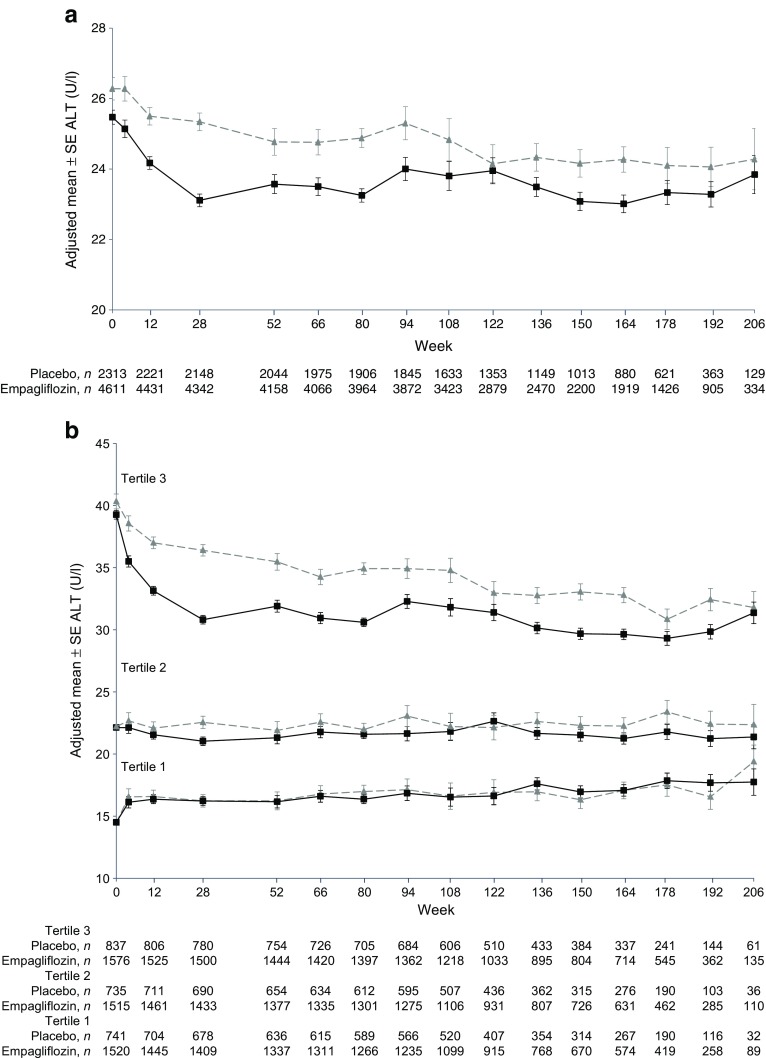
Changes in ALT in the EMPA-REG OUTCOME^**®**^ trial. (**a**) All patients. Adjusted mean ± SE change from baseline: −0.73 ± 0.25 with placebo and −2.96 ± 0.18 with empagliflozin at week 28; −1.80 ± 0.36 with placebo and −3.06 ± 0.25 with empagliflozin at week 164. (**b**) Patients in tertiles by baseline ALT. Adjusted mean ± SE change from baseline at week 28: 1.57 ± 0.51 with placebo and 1.51 ± 0.36 with empagliflozin in tertile 1; 0.29 ± 0.51 with placebo and −1.15 ± 0.35 with empagliflozin in tertile 2; −3.96 ± 0.48 with placebo and −8.33 ± 0.35 with empagliflozin in tertile 3. Adjusted mean ± SE change from baseline at week 164: 2.46 ± 0.72 with placebo and 2.32 ± 0.49 with empagliflozin in tertile 1; 0.04 ± 0.71 with placebo and −0.86 ± 0.48 with empagliflozin in tertile 2; −7.33 ± 0.65 with placebo and −9.54 ± 0.46 with empagliflozin in tertile 3. MMRM analysis in patients treated with at least one dose of study drug based on observed cases, including values after initiation of rescue medication. Baseline values are mean ± SE. Grey triangles and dashed lines, placebo; black squares and solid lines, empagliflozin

In the EMPA-REG OUTCOME^**®**^ trial, ALT decreased from baseline to week 28 and thereafter remained stable in the empagliflozin group, and declined steadily and to a lesser extent in the placebo group (Fig. 1a). Mean ± SE changes from baseline were −2.96 ± 0.18 U/l in the empagliflozin group and −0.73 ± 0.25 in the placebo group at week 28 (adjusted mean difference: −2.22 [95% CI −2.83, −1.62]; *p* < 0.0001) and −3.06 ± 0.25 U/l and −1.80 ± 0.36 U/l, respectively, at week 164 (adjusted mean difference: −1.26 [95% CI −2.12, −0.40]; *p* = 0.0040) (Table 1).

**Table 1:** Differences in changes from baseline ALT and AST with empagliflozin relative to placebo or glimepiride

Trial	ALT (U/l)	AST (U/l)
EMPA-REG OUTCOME^**®**^		
Adjusted mean (95% CI) difference vs placebo in change from baseline at week 28		
All participants	−2.22 (−2.83, −1.62) *p* < 0.0001	−1.01 (−1.48, −0.54) *p* < 0.0001
Tertile 3 (highest tertile) by ALT/AST at baseline	−4.36 (−5.51, −3.21) *p* < 0.0001	−1.53 (−2.35, −0.72) *p* = 0.0002
Adjusted mean (95% CI) difference vs placebo in change from baseline at week 164		
All participants	−1.26 (−2.12, −0.40) *p* = 0.0040	−0.66 (−1.45, 0.13) *p* = 0.1007
Tertile 3 (highest tertile) by ALT/AST at baseline	−2.22 (−3.78, −0.66) *p* = 0.0053	−0.99 (−2.34, 0.36) *p* = 0.1504
Pooled 24-week placebo-controlled trial data		
Adjusted mean (95% CI) difference vs placebo in change from baseline at week 24		
All participants	−3.15 (−4.11, −2.18) *p* < 0.0001	−1.45 (−2.12, −0.78) *p* < 0.0001
Tertile 3 (highest tertile) by ALT/AST at baseline	−5.60 (−7.41, −3.79) *p* < 0.0001	−2.84 (−4.14, −1.54) *p* < 0.0001
EMPA-REG H2H-SU		
Adjusted mean (95% CI) difference vs glimepiride in change from baseline at week 28		
All participants	−4.88 (−6.68, −3.09) *p* < 0.0001	−2.78 (−3.92, −1.64) *p* < 0.0001
Tertile 3 (highest tertile) by ALT/AST at baseline	−7.14 (−10.11, −4.18) *p* < 0.0001	−3.92 (−5.79, −2.04) *p* < 0.0001
Adjusted mean (95% CI) difference vs glimepiride in change from baseline at week 104		
All participants	−4.28 (−6.11, −2.45) *p* < 0.0001	−3.00 (−4.33, −1.67) *p* < 0.0001
Tertile 3 (highest tertile) by ALT/AST at baseline	−6.03 (−9.02, −3.04) *p* < 0.0001	−5.25 (−7.47, −3.03) *p* < 0.0001

MMRM analysis in participants treated with at least one dose of study drug based on observed cases, including values after initiation of rescue medication

**Fig. 2:**
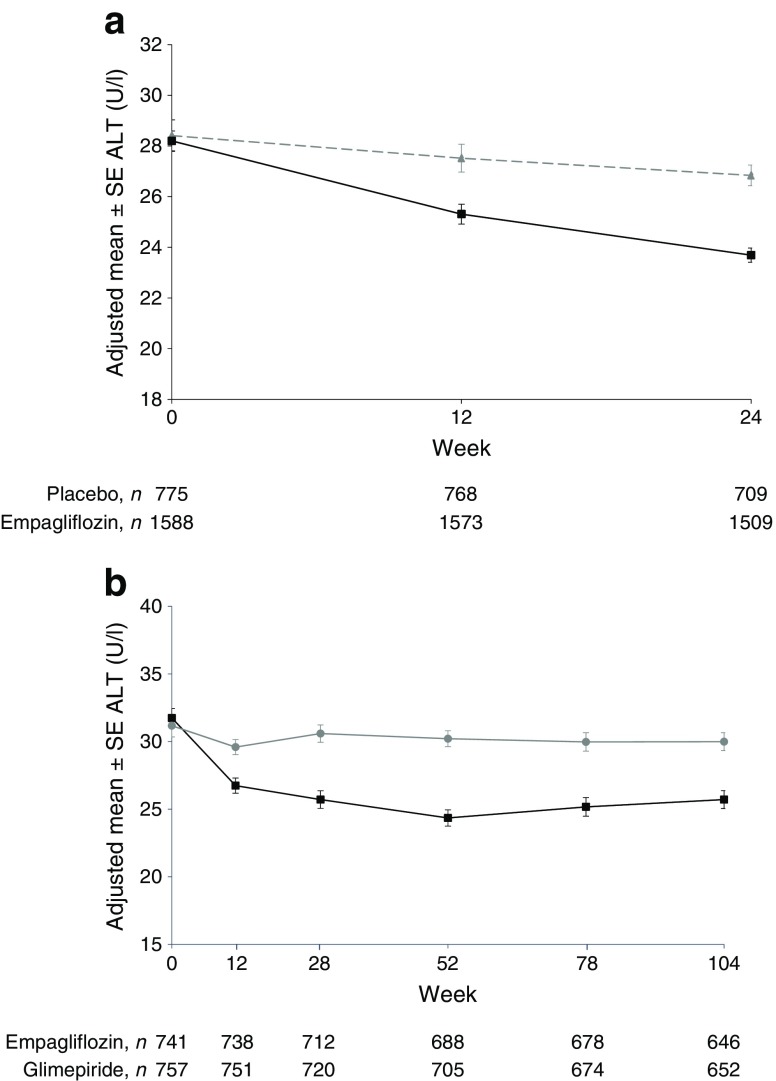
(**a**) Changes in ALT in pooled 24 week trial data. Adjusted mean ± SE change from baseline at week 24: −1.40 ± 0.41 with placebo and −4.55 ± 0.28 with empagliflozin (grey triangles and dashed line, placebo; black squares and solid line, empagliflozin [25 mg]). (**b**) Changes in ALT in the EMPA-REG H2H-SU trial. Adjusted mean ± SE change from baseline: −0.77 ± 0.64 with glimepiride and −5.65 ± 0.65 with empagliflozin at week 28; −1.37 ± 0.66 with glimepiride and −5.65 ± 0.66 with empagliflozin at week 104 (black squares and line, empagliflozin [25 mg]; grey circles and line, glimepiride). MMRM analysis in patients treated with at least one dose of study drug based on observed cases, including values after initiation of rescue medication. Baseline values are mean ± SE

Reductions in ALT were greatest in participants in the highest tertile of ALT at baseline (Fig. 1b), in whom the adjusted mean difference with empagliflozin vs placebo was −4.36 U/l (95% CI −5.51, −3.21; *p* < 0.0001) at week 28 and −2.22 U/l (95% CI −3.78, −0.66; *p* = 0.0053) at week 164 (Table 1). In the second tertile, differences with empagliflozin vs placebo in changes from baseline in ALT were −1.45 (95% CI −2.65, −0.24; *p* = 0.0188) at week 28 and −0.91 (95% CI −2.59, 0.77; *p* = 0.2903) at week 164. In the lowest tertile, changes from baseline in ALT were similar between empagliflozin and placebo at weeks 28 and 164 (Fig. 1b). Similar patterns of changes in ALT were observed with empagliflozin vs placebo in the pooled 24-week trial data (Fig. 2a and electronic supplementary material [ESM] Fig. 1), and with empagliflozin vs glimepiride over 104 weeks in the EMPA-REG H2H-SU trial (Fig. 2b, ESM Fig. 2).

In the EMPA-REG OUTCOME^**®**^ trial, treatment differences in ALT were largely independent of concomitant changes in HbA_1c_ or weight in the overall trial population (Table 2).

**Table 2:** Proportions of changes in ALT mediated by changes in HbA_1c_ and weight in the EMPA-REG OUTCOME^**®**^ trial

Proportion of ALT-lowering effect of empagliflozin vs placebo that was independent of/associated with concomitant changes from baseline in HbA_1c_ or weight	Independent (%)	Associated (%)
Week 28		
Change from baseline in HbA_1c_	88.0	12.0
Change from baseline in weight	86.8	13.2
Change from baseline in HbA_1c_ and weight	76.7	23.3
Week 52		
Change from baseline in HbA_1c_	82.6	17.4
Change from baseline in weight	79.0	21.0
Change from baseline in HbA_1c_ and weight	66.2	33.8
Week 164		
Change from baseline in HbA_1c_	85.0	15.0
Change from baseline in weight	90.6	9.4
Change from baseline in HbA_1c_ and weight	76.7	23.3

MMRM analysis in participants treated with at least one dose of study drug based on observed cases, including values after initiation of rescue medication

HbA_1c_: *n* analysed in placebo and empagliflozin groups, respectively = 2128 and 4308, at week 28; 2025 and 4123 at week 52; and 874 and 1904 at week 164

Weight: *n* analysed in placebo and empagliflozin groups, respectively = 2140 and 4336 at week 28; 2037 and 4146 at week 52; and 876 and 1907 at week 164

### Changes in AST

In the EMPA-REG OUTCOME^**®**^ trial, AST decreased from baseline to week 28 and thereafter remained stable in the empagliflozin group, while there were no relevant changes in the placebo group (ESM Fig. 3a). Mean ± SE changes from baseline were −1.32 ± 0.14 U/l in the empagliflozin group and −0.31 ± 0.20 in the placebo group at week 28 (adjusted mean difference: −1.01 U/l [95% CI −1.48, −0.54]; *p* < 0.0001), and −1.17 ± 0.23 U/l and −0.51 ± 0.33 U/l, respectively at week 164 (adjusted mean difference: −0.66 U/l [95% CI −1.45, 0.13]; *p* = 0.1007) (Table 1). Reductions from baseline in AST with empagliflozin were greatest in participants in the highest tertile of AST at baseline (ESM Fig. 3b), in whom the adjusted mean difference with empagliflozin vs placebo was −1.53 U/l (95% CI −2.35, −0.72; *p* = 0.0002) at week 28 and −0.99 U/l (95% CI −2.34, 0.36; *p* = 0.1504) at week 164 (Table 1). Changes from baseline AST were similar between empagliflozin and placebo at weeks 28 and 164 in the second and third tertiles (ESM Fig. 3b). Similar patterns of changes in AST were observed with empagliflozin vs placebo in the pooled 24-week trial data (ESM Fig. 4), and with empagliflozin vs glimepiride over 104 weeks in the EMPA-REG H2H-SU trial (ESM Fig. 5). In all studies examined, while not compared statistically, changes in AST were of a substantially lower magnitude than changes in ALT (Table 1).

## Discussion

Type 2 diabetes is often associated with NAFLD, which is reflected by increased liver aminotransferase levels. ALT levels above the upper limit of normal and AST levels lower than those of ALT are typical findings supporting the presence of NAFLD in real-life clinical practice in primary care [4]. Our analyses from three large datasets from clinical trials of empagliflozin in individuals with type 2 diabetes have shown that treatment with empagliflozin consistently lowered ALT and AST levels. The reductions in ALT observed with empagliflozin were consistently greater than the reductions in AST, more prominent in participants with the highest ALT levels at baseline and largely independent of changes in HbA_1c_ and weight. Changes in ALT and AST were minimal in participants with the lowest levels at baseline, who would be predicted to have the lowest liver fat levels.

Transaminases, in particular ALT, are useful correlates of liver fat. The overall patterns of change in ALT observed with empagliflozin are broadly consistent with a reduction in liver fat [4]. The twin-cycle hypothesis of diabetes postulates that excess liver and pancreatic fat are major components of the pathogenesis of type 2 diabetes [25]. Furthermore, it has recently been shown that, among individuals with type 2 diabetes, those with a hospital record of NAFLD have an increased risk of all-cause mortality, death from cardiovascular disease and incident/recurrent cardiovascular events than those without a record of liver disease [26]. Hence, any treatment that lowers liver fat and the risk of progression to more advanced liver disease could have important benefits for individuals with type 2 diabetes.

The mechanisms by which empagliflozin might reduce aminotransferases or liver fat are unclear. Data from animal models support a direct effect of empagliflozin on reducing liver fat and improving hepatic glucose handling. In *db/db* mice, glucose uptake in the liver and kidneys has been reported to be higher in mice treated with empagliflozin than in controls [27], and in a NASH model generated by administration of streptozotocin to C57BL/6J mice, empagliflozin attenuated the development of NASH via anti-steatotic and anti-inflammatory effects [28]. The SGLT2 inhibitor dapagliflozin has also been shown to improve markers of liver fat in individuals with type 2 diabetes and NASH [29]. Further emerging evidence, albeit from a much smaller trial, has linked treatment with other SGLT2 inhibitors to reductions in liver fat (and ALT) equivalent to those seen with pioglitazone in individuals with type 2 diabetes [30].

Liver fat was not measured directly in any of the studies used in our analyses. However, in a study using magnetic resonance spectroscopy, ALT levels were found to correlate reasonably well with the extent of liver fat in individuals with type 2 diabetes (*r* = 0.66), even though liver fat levels were disproportionately (200%) higher in those with type 2 diabetes than in control participants without diabetes at given ALT levels in the range of 50–200 U/l [9]. This suggests that in individuals with type 2 diabetes, reductions in ALT from higher baseline levels (equivalent to the top ALT tertile in our analyses) are more likely to be associated with meaningful reductions in liver fat. In the current analysis, the patterns of reduction, the greater effects observed when baseline ALT was higher and the consistent observations across three large datasets lend confidence that our findings reflect a real clinical action of empagliflozin. However, we accept that further trials are needed, including those that take histological measures of NASH in individuals with type 2 diabetes before and after treatment. Ongoing trials, including long-term trials of empagliflozin in individuals with heart failure (NCT03057977, NCT03057951) and chronic kidney disease [31], will provide additional data.

We also accept that ALT levels at baseline were measured at only a single point in time and that a sustained elevation in ALT is potentially a better marker of a higher level of liver fat. While overall reductions in ALT were modest, reductions with empagliflozin relative to placebo or sulfonylurea were in the order of 4–7 U/l in those with higher ALT levels at baseline, which appears meaningful. Finally, of course, we accept that other conditions that influence the liver, such as hepatitis or excess alcohol intake, can influence ALT and AST levels, although alcohol does so in a different pattern [4]. Furthermore, it is unlikely that the greater changes in ALT that we observed with empagliflozin vs placebo could occur via effects on subclinical hepatitis, which would be present in only a minority of individuals.

In conclusion, our results suggest that in individuals with type 2 diabetes, treatment with empagliflozin reduces liver aminotransferases, largely independent of changes in weight and glycaemic control, and does so in a pattern that is potentially consistent with reductions in liver fat. These highly consistent trial findings add meaningfully to the emerging evidence from smaller trials on the effects of SGLT2 inhibitors on direct measures of liver fat, and collectively suggest a potential role for this class of drugs in managing NAFLD in type 2 diabetes. Further trials should now be promoted to enable an indication to be developed.

## Electronic supplementary material

ESM Figures (PDF 236 kb)

## Data Availability

The datasets generated during and/or analysed during the current study are available from the corresponding author on reasonable request.

## Abbreviations

ALT: Alanine aminotransferase
AST: Aspartate aminotransferase
MDRD: Modification of Diet in Renal Disease
MMRM: Mixed-model repeated measures
NAFLD: Non-alcoholic fatty liver disease
NASH: Non-alcoholic steatohepatitis
SGLT2: Sodium–glucose cotransporter 2
