# Supplementary material for: Empagliflozin is associated with improvements in liver enzymes potentially consistent with reductions in liver fat: results from randomised trials including the EMPA-REG OUTCOME® trial
Source: Diabetologia. 2018 Jul 31;61(10):2155–63. doi: 10.1007/s00125-018-4702-3 (PMC6133166; doi:10.1007/s00125-018-4702-3)
Supplement: Supplementary file 1 — (PDF 236 kb) [file 125_2018_4702_MOESM1_ESM.pdf]

Empagliflozin is associated with improvements in liver enzymes potentially consistent with reductions in liver fat: results from randomised trials including the EMPA-REG OUTCOME® trial (Sattar N et al.)

Electronic supplementary material (ESM)

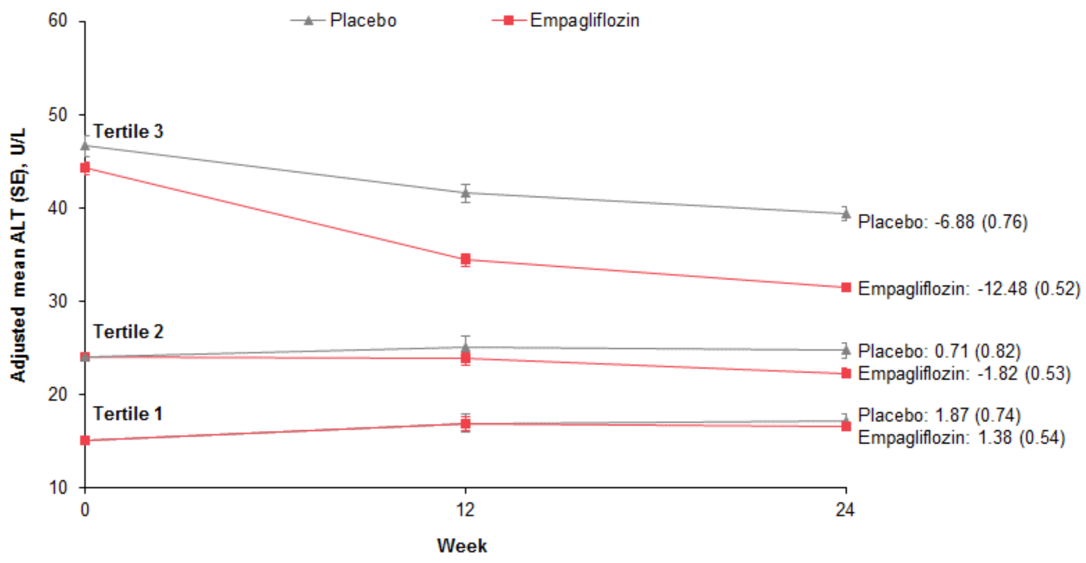

**Tertile 3**			
Placebo (n)	286	283	260
Empagliflozin (n)	515	510	487
**Tertile 2**			
Placebo (n)	228	227	207
Empagliflozin (n)	519	514	499
**Tertile 1**			
Placebo (n)	261	258	242
Empagliflozin (n)	554	549	523

ESM Figure 1. Changes in alanine aminotransferase (ALT) in pooled 24-week trial data in tertiles by baseline ALT. Mixed model repeated measures analysis in patients treated with ≥1 dose of study drug based on observed cases, including values after initiation of rescue medication. Baseline values are mean (SE). SE, standard error.

a

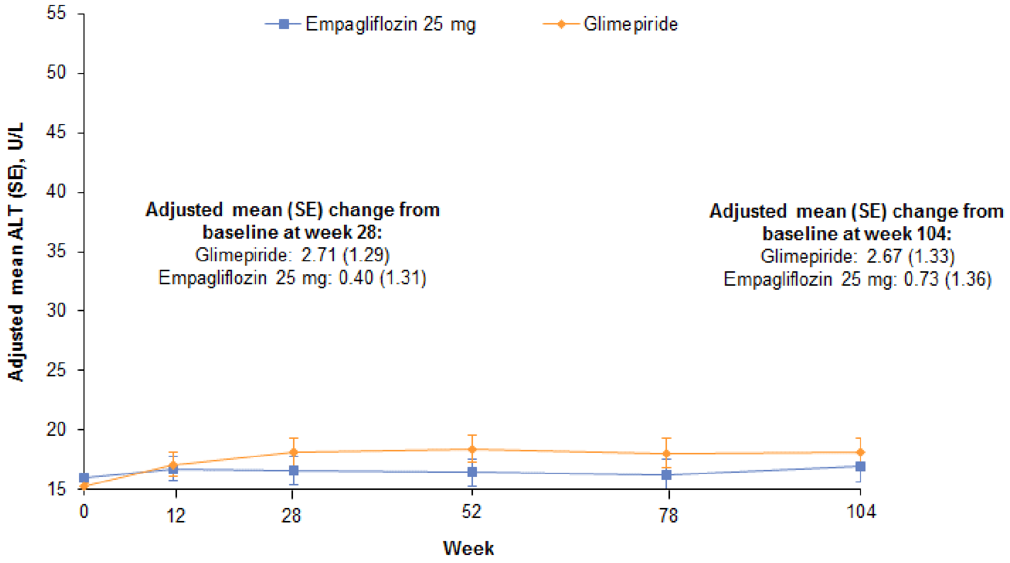

Glimepiride (n)	246	243	234	229	222	216
Empagliflozin 25 mg (n)	238	236	231	216	217	203

b

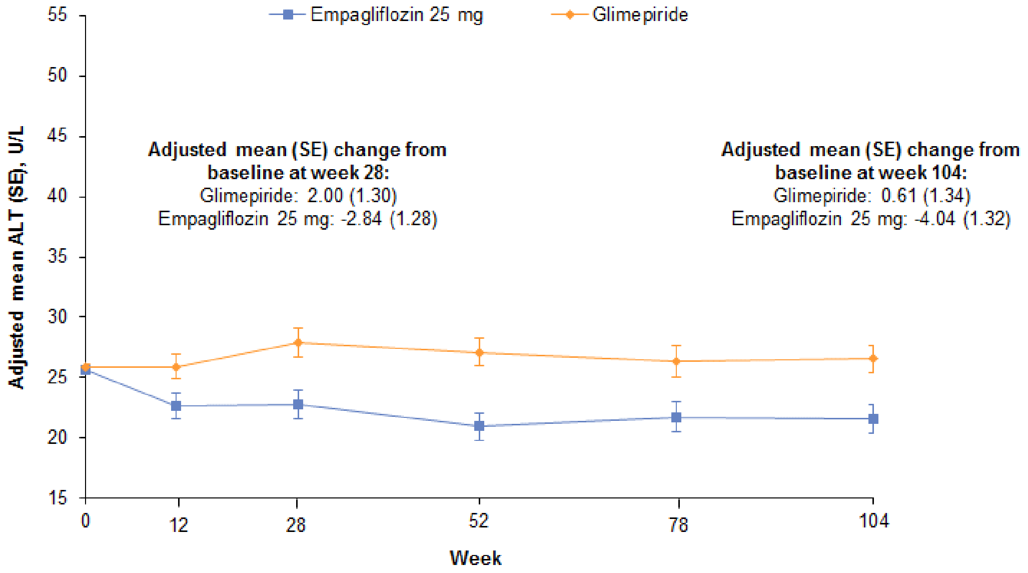

Glimepiride (n)	240	239	229	227	219	211
Empagliflozin 25 mg (n)	248	248	235	231	227	219

c

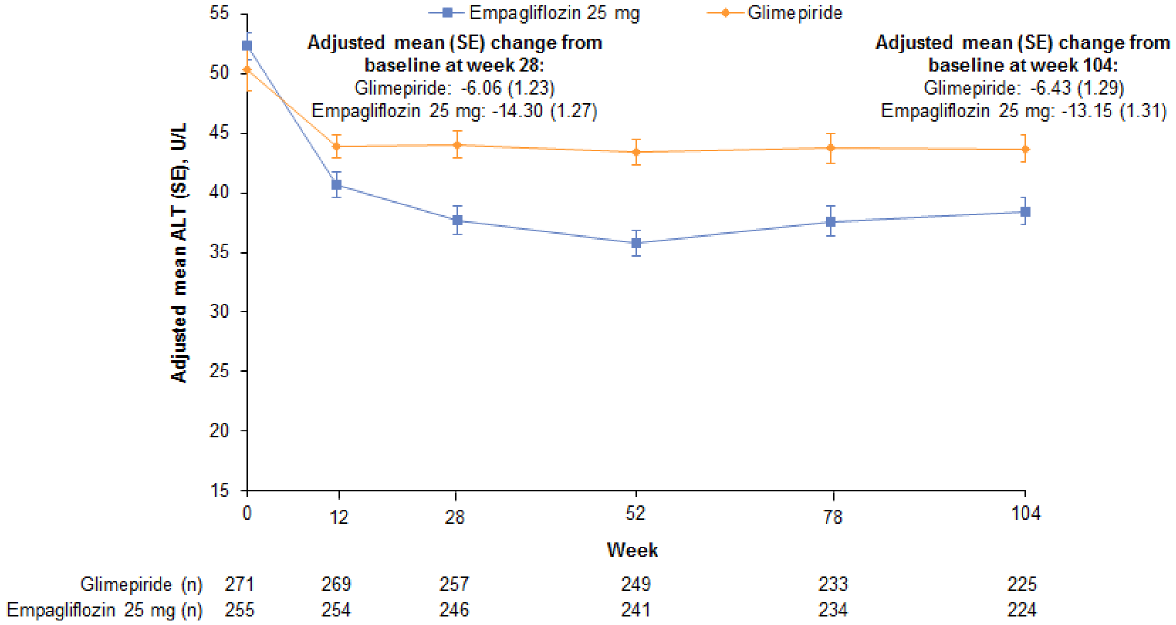

ESM Figure 2. Changes in alanine aminotransferase (ALT) in the EMPA-REG H2H-SU trial in tertiles of patients by baseline ALT: (**a**) tertile 1; (**b**) tertile 2; (**c**) tertile 3. Mixed model repeated measures analysis in patients treated with ≥1 dose of study drug based on observed cases, including values after initiation of rescue medication. Baseline values are mean (SE). SE, standard error.

a

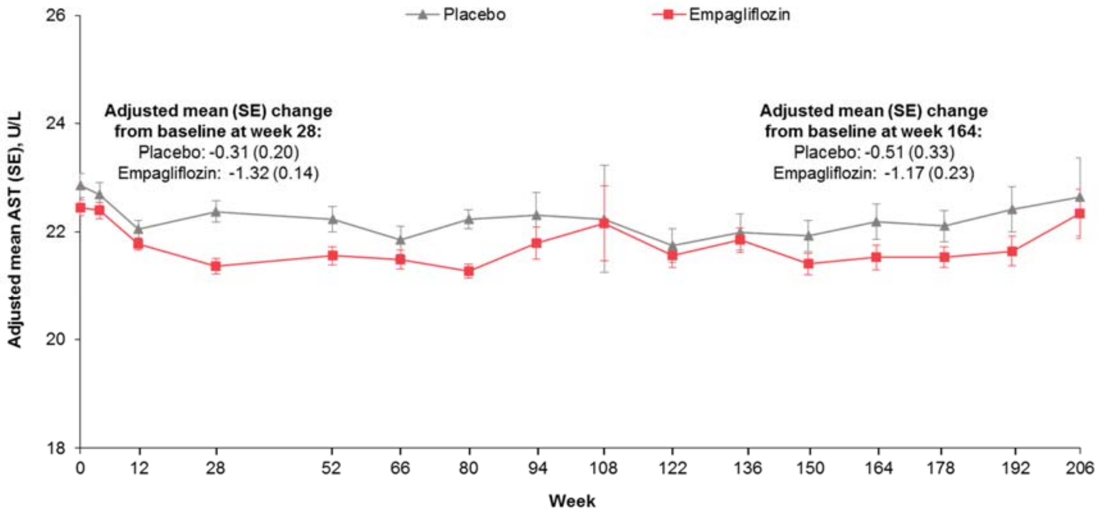

Placebo (n)	2312	2217	2146	2041	1973	1902	1844	1629	1351	1146	1009	877	617	362	129
Empagliflozin (n)	4611	4426	4330	4154	4058	3957	3865	3418	2876	2466	2195	1912	1425	905	330

b

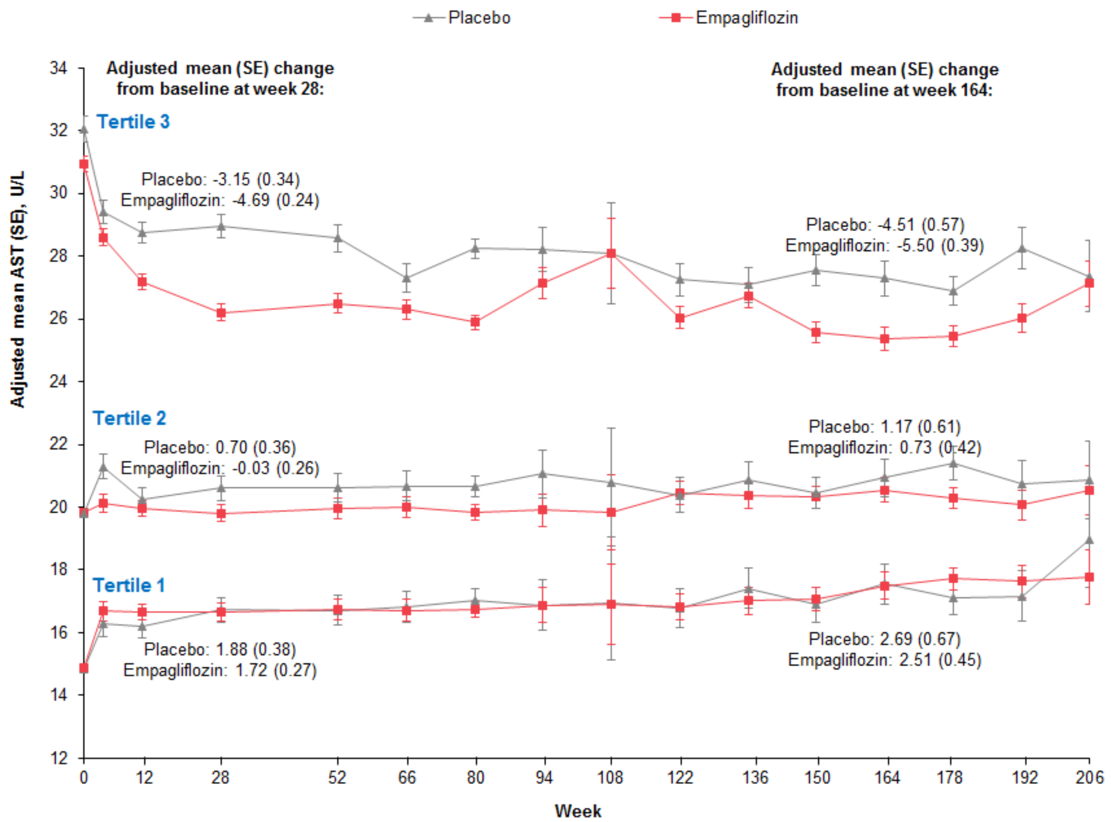

**Tertile 3**															
Placebo (n)	861	835	805	773	743	713	696	610	514	431	384	336	243	147	53
Empagliflozin (n)	1713	1647	1619	1551	1527	1491	1456	1290	1088	944	836	737	545	357	129
**Tertile 2**															
Placebo (n)	757	719	706	665	642	629	604	529	447	385	336	299	200	111	46
Empagliflozin (n)	1487	1430	1406	1351	1328	1293	1263	1131	968	823	745	635	482	289	109
**Tertile 1**															
Placebo (n)	694	663	635	603	588	560	544	490	390	330	289	242	174	104	30
Empagliflozin (n)	1411	1349	1305	1252	1203	1173	1146	997	820	699	614	540	398	259	92

ESM Figure 3. Changes in aspartate aminotransferase (AST) in the EMPA-REG OUTCOME® trial in (**a**) all patients and (**b**) tertiles by baseline AST. Mixed model repeated measures analysis in patients treated with ≥1 dose of study drug based on observed cases, including values after initiation of rescue medication.

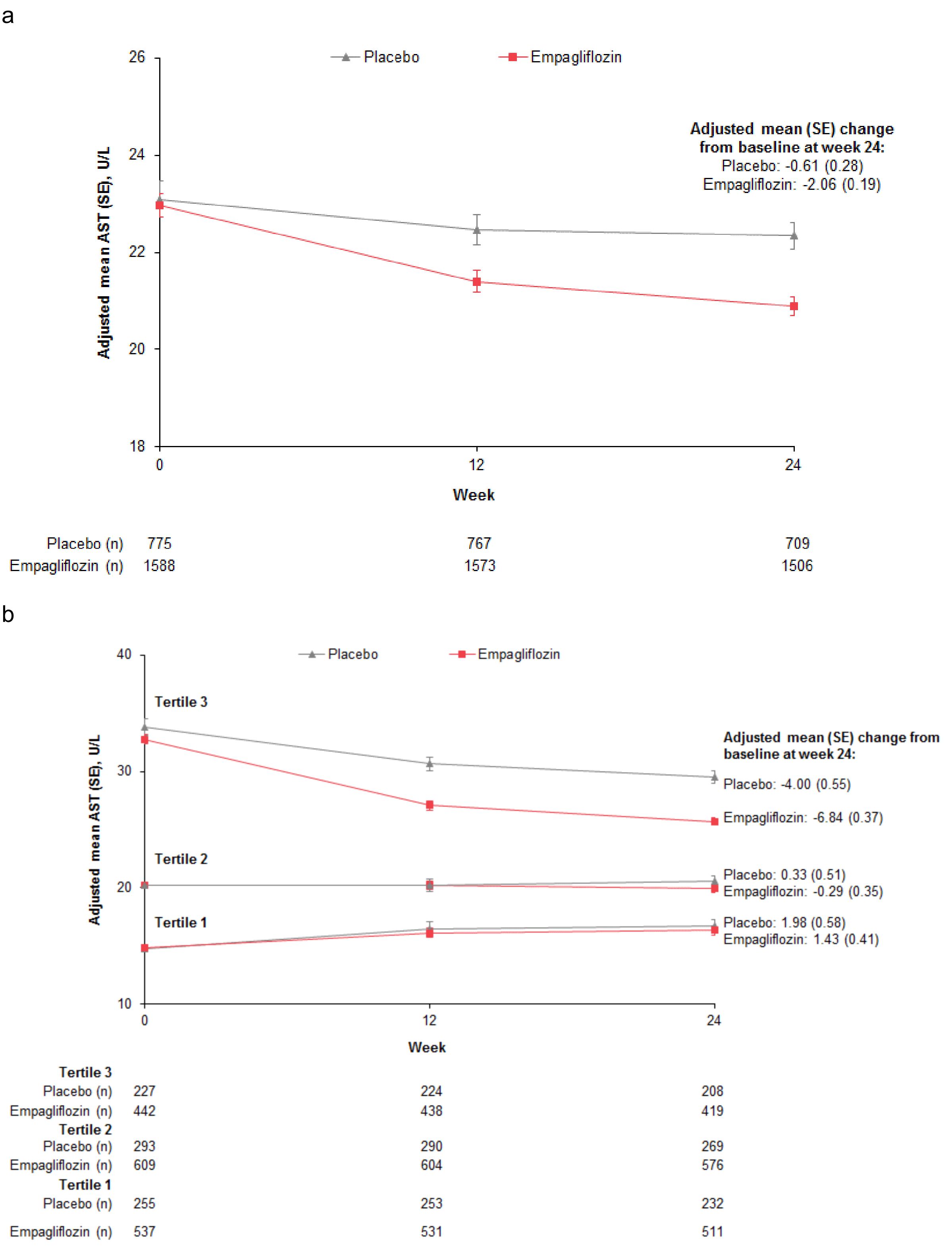

ESM Figure 4. Changes in aspartate aminotransferase (AST) in pooled 24-week trial data in (**a**) all patients and (**b**) tertiles of patients by baseline ALT. Mixed model repeated measures analysis in patients treated with ≥1 dose of study drug based on observed cases, including values after initiation of rescue medication. Baseline values are mean (SE). SE, standard error.

a

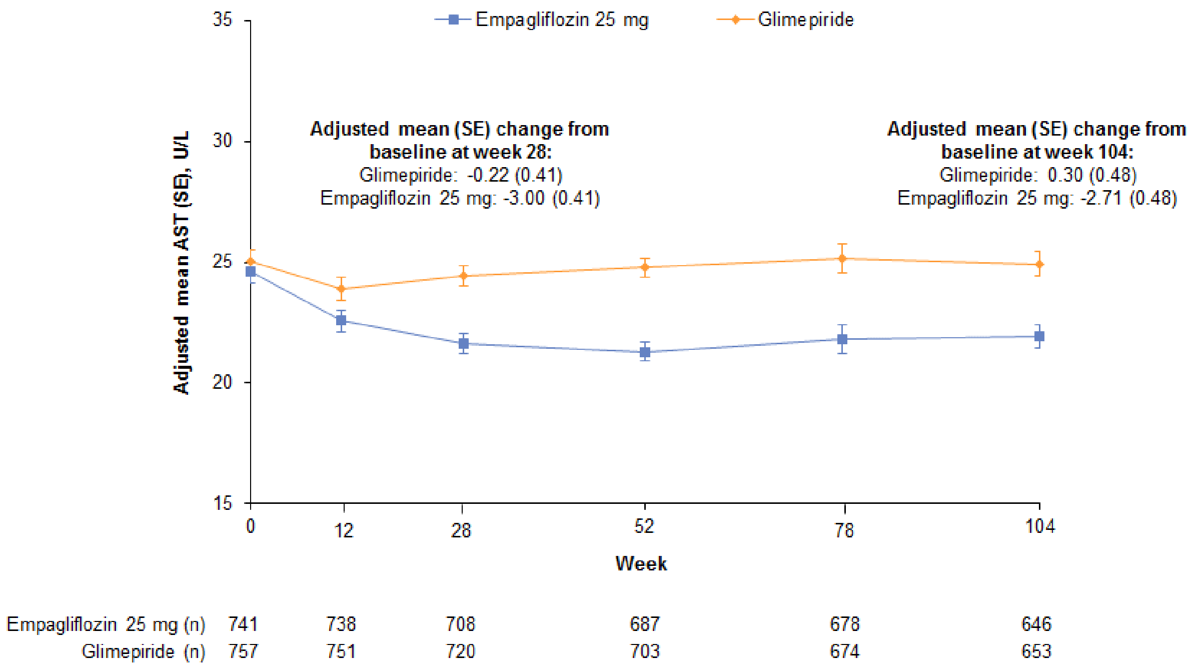
Empagliflozin 25 mg
Glimepiride
Adjusted mean (SE) change from baseline at week 28:
Glimepiride: -0.22 (0.41)
Empagliflozin 25 mg: -3.00 (0.41)
Adjusted mean (SE) change from baseline at week 104:
Glimepiride: 0.30 (0.48)
Empagliflozin 25 mg: -2.71 (0.48)
Adjusted mean AST (SE), U/L
35
30
25
20
15
0
12
28
52
78
104
Week
Empagliflozin 25 mg (n) 741 738 708 687 678 646
Glimepiride (n) 757 751 720 703 674 653

b

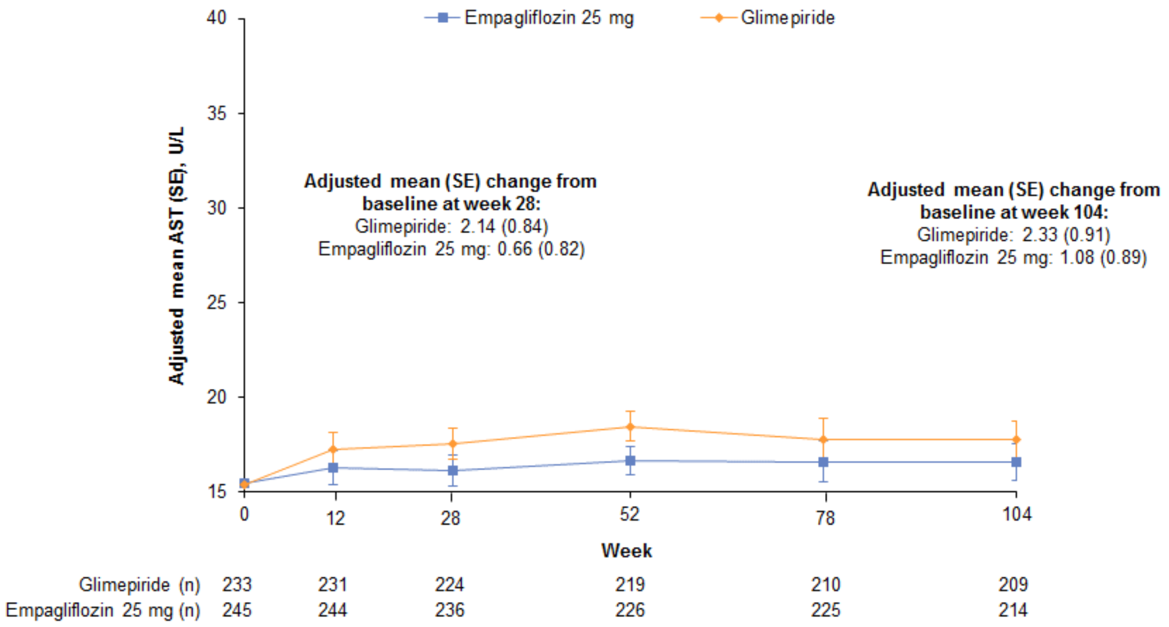
Empagliflozin 25 mg
Glimepiride
Adjusted mean (SE) change from baseline at week 28:
Glimepiride: 2.14 (0.84)
Empagliflozin 25 mg: 0.66 (0.82)
Adjusted mean (SE) change from baseline at week 104:
Glimepiride: 2.33 (0.91)
Empagliflozin 25 mg: 1.08 (0.89)
Adjusted mean AST (SE), U/L
40
35
30
25
20
15
0
12
28
52
78
104
Week
Glimepiride (n) 233 231 224 219 210 209
Empagliflozin 25 mg (n) 245 244 236 226 225 214

c

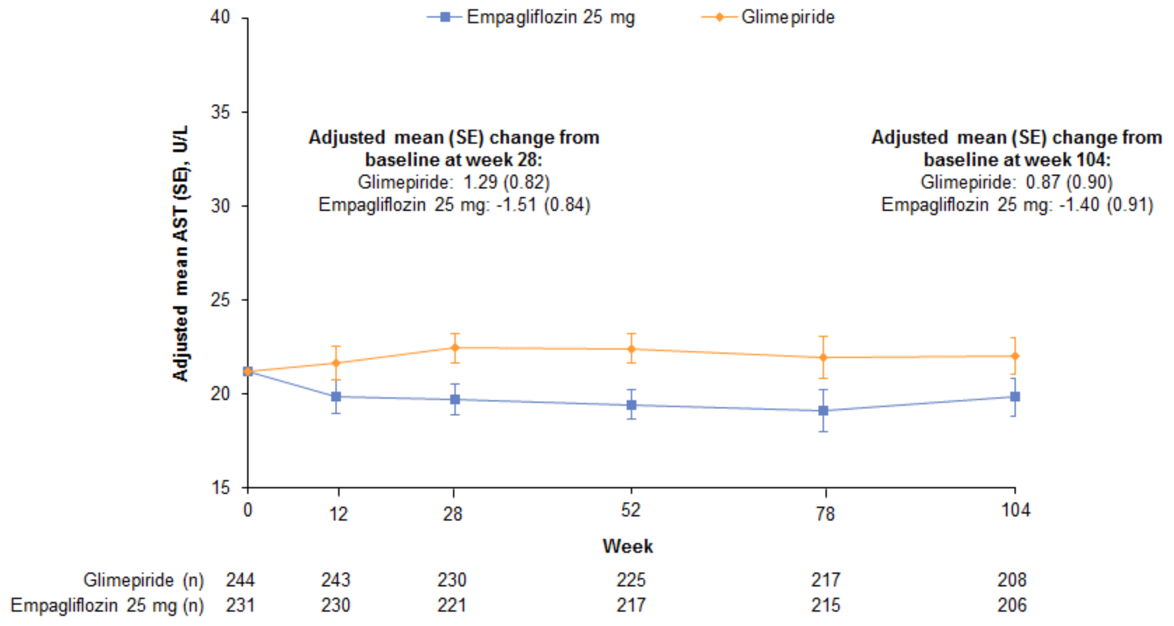

d

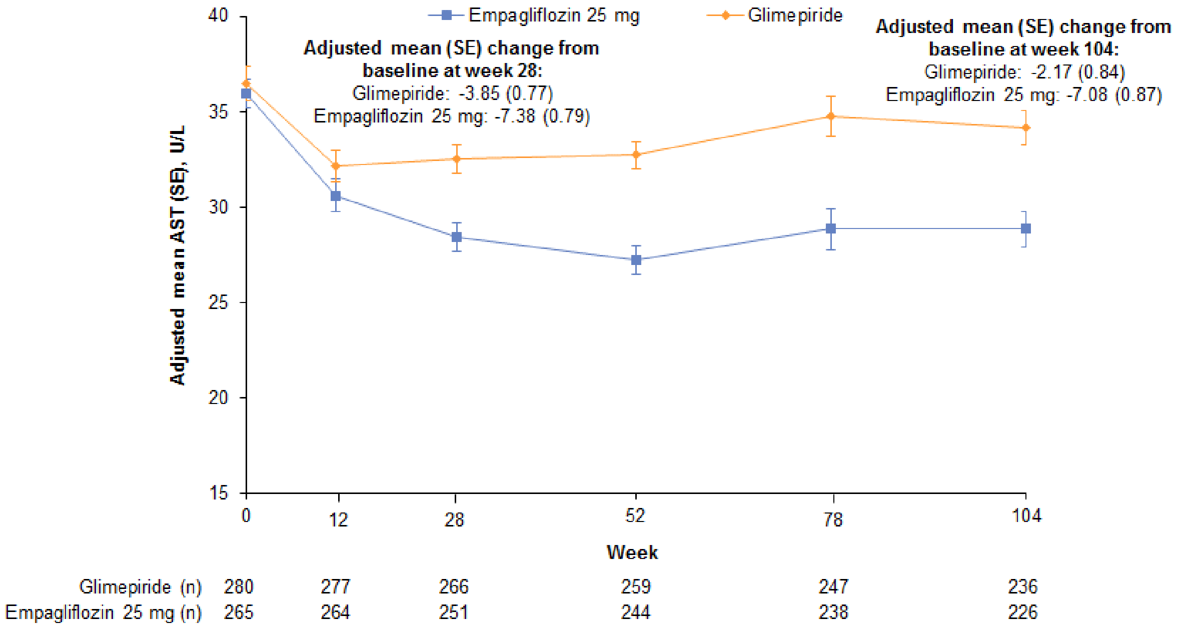

ESM Figure 5. Changes in aspartate aminotransferase (AST) in the EMPA-REG H2H-SU trial in (**a**) all patients and in tertiles of patients by baseline ALT: (**b**) tertile 1; (**c**) tertile 2; (**d**) tertile 3. Mixed model repeated measures analysis in patients treated with ≥1 dose of study drug based on observed cases, including values after initiation of rescue medication. Baseline values are mean (SE). SE, standard error
